# Cryptic *KMT2A::AFDN* Fusion Due to *AFDN* Insertion into *KMT2A* in a Patient with Acute Monoblastic Leukemia

**DOI:** 10.3390/genes16030317

**Published:** 2025-03-07

**Authors:** Qing Wei, Gokce A. Toruner, Beenu Thakral, Keyur P. Patel, Naveen Pemmaraju, Sa A. Wang, Rashmi Kanagal-Shamanna, Guilin Tang, Ghayas C. Issa, Sanam Loghavi, L Jeffrey Medeiros, Courtney DiNardo

**Affiliations:** 1 Department of Hematopathology, The University of Texas MD Anderson Cancer Center, 6565 MD Anderson Avenue, Z5.5048, Houston, TX 77030, USA; qwei1@mdanderson.org (Q.W.); bthakral@mdanderson.org (B.T.); kppatel@mdanderson.org (K.P.P.); swang5@mdanderson.org (S.A.W.); rkanagal@mdanderson.org (R.K.-S.); gtang@mdanderson.org (G.T.); sloghavi@mdanderson.org (S.L.); ljmedeiros@mdanderson.org (L.J.M.); 2 Department of Leukemia, The University of Texas MD Anderson Cancer Center, 6565 MD Anderson Avenue, Z5.5048, Houston, TX 77030, USA; npemmaraju@mdanderson.org (N.P.); gcissa@mdanderson.org (G.C.I.); cdinardo@mdanderson.org (C.D.)

**Keywords:** *KMT2A::AFDN* rearrangement, fluorescence in situ hybridization (FISH), RNA fusion panel, optical genome mapping, acute monocytic leukemia

## Abstract

Background: *KMT2A* rearrangements occur in ~10% of acute myeloid leukemia (AML) cases and are critical for classification, risk stratification, and use of targeted therapy. However, insertions involving the *KMT2A* gene can evade detection using chromosomal analysis and/or fluorescence in situ hybridization (FISH). Methods: We present a case of a 22-year-old woman with acute monoblastic leukemia harboring a cryptic *KMT2A::AFDN* fusion identified by RNA sequencing. Initial FISH showed a 3′ *KMT2A* deletion, while conventional karyotyping and the automated bioinformatic pipeline for optical genome mapping (OGM) did not identify the canonical translocation. Results: To resolve these discrepancies, metaphase *KMT2A* FISH (break-apart fusion probe) was performed to assess whether *KMT2A* was translocated to another chromosome. However, the results did not support this possibility. As the fusion signal remained on the normal chromosome 11, with the 5′ *KMT2A* signal localized to the derivative chromosome 11. A subsequent manual review of the OGM data revealed a cryptic ~300 kb insertion of *AFDN* into the 3′ region of *KMT2A*, reconciling the discrepancies between chromosomal analysis, FISH, and RNA fusion results. Conclusions: This case highlights the importance of integrating multiple testing modalities with expert review when there is a discrepancy. Our findings emphasize the need for a comprehensive approach to genomic assessment to enhance diagnostic accuracy and guide therapeutic decision-making.

## 1. Introduction

*KMT2A* rearrangements are identified in approximately 10% of cases of acute myeloid leukemia (AML) that occur across all age groups and represent distinct genetically defined types of AML. *KMT2A* (lysine methyltransferase 2A), formerly known as *MLL*, has been shown to have over 100 identified fusion partners [1]. Among these, the seven most frequent fusion partners—*MLLT3* (30%), *MLLT10* (19%), ELL (10%), AFDN (8%), *MLLT1* (4%), *EPS15* (1%), AFF1 (1%), and partial tandem duplication (10%)—account for approximately 80% of *KMT2A* recombination events in AML [1]. *KMT2A* rearranged AML is associated with a poor prognosis, with a notable exception of t(9;11)(p21;q23)/*KMT2A::MLLT3*, which confers an intermediate prognosis [2]. *KMT2A* encodes a histone methyltransferase that regulates gene expression, and its rearrangements result in the upregulation of *HOX* family genes, driving leukemogenesis via arrested differentiation [3]. Targeting the KMT2A menin complex with menin inhibitors has been shown to be efficacious in reversing this arrest in differentiation. Fusion partners such as *ABI1*, *AFDN*, *AFF1*, *MLLT1*, and *MLLT10* are associated with a higher risk of relapse, while other partners, including *ELL*, *MLLT3*, *MLLT11*, and *SEPTIN6*, are associated with standard risk [4]. Detecting *KMT2A* rearrangements is critical for disease classification, risk stratification, and therapeutic decision-making, as these rearrangements are targetable using menin inhibitors [5]. The menin inhibitor revumenib was recently approved for the treatment of relapsed/refractory acute leukemias with *KMT2A* rearrangements by the US Food and Drug Administration.

In many cases, detecting *KMT2A* rearrangements requires integrating multiple testing modalities. *KMT2A* rearrangement can escape detection when using conventional karyotyping techniques and the rearrangement may present as a simple deletion of chromosome 11/del(11)(q23), or as cryptic rearrangements with insertions or subtle breakpoints [1,6,7,8,9,10]. The *KMT2A* fluorescence in situ hybridization (FISH) break-apart probe is widely used in clinical laboratories to detect *KMT2A* rearrangements because this gene has numerous fusion partners, but this approach has limitations, including the inability to detect cryptic fusions, such as *KMT2A::USP2* [7], or insertions [8,9], or partial tandem duplication [10], and FISH also does not provide information regarding specific fusion partners. Next-generation sequencing (NGS)-based RNA fusion panels provide higher resolution and can identify fusion partners and junctions with precision. However, these assays do not provide insights into underlying DNA alterations, especially complex rearrangements. Optical genome mapping (OGM) is a non-sequencing-based, whole-genome screening technology that detects complex structural variants with high resolution but cannot identify precise nucleotide-level junctions. Therefore, each testing modality has limitations, and discrepancies generated using these methods can present diagnostic challenges.

Here, we present a case of acute monoblastic leukemia in which different testing methods yielded discrepant *KMT2A* rearrangement results. Archer RNA fusion analysis identified *KMT2A::AFDN* fusion transcripts, and *KMT2A* FISH analysis showed a 3′ deletion suggestive of unbalanced *KMT2A* rearrangement. However, conventional chromosomal analysis and an initial analysis using our automated bioinformatic pipeline for OGM did not identify t(6;11)(q27;q23)/*KMT2A::AFDN*.

Due to our knowledge of the RNA sequencing result, we performed an additional manual inspection of OGM results at the *KMT2A* locus and identified a cryptic insertion of *AFDN* into the 3′ region of *KMT2A*, explaining the discrepant results. The RNA fusion was the result of an intragenic insertion event rather than an interchromosomal translocation.

## 2. Case Report

**Figure 1:**
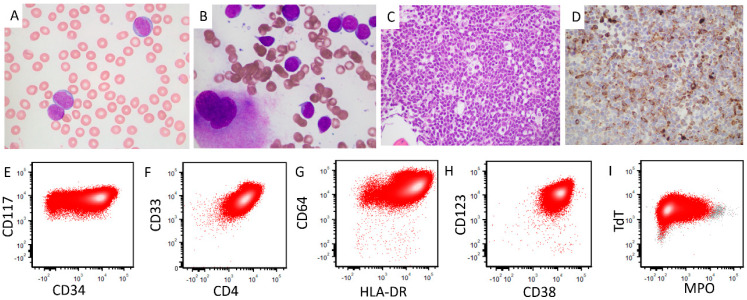
Morphologic and immunophenotypic findings in peripheral blood and bone marrow specimens. (**A**,**B**): Peripheral blood (**A**) and bone marrow aspirate (**B**) smears show large blasts with open chromatin, variably conspicuous nucleoli, round to indented nuclear membranes, and moderate basophilic cytoplasm. No Auer rods were identified (×1000). (**C**): The bone marrow biopsy specimen shows a hypercellular bone marrow with sheets of large blasts displaying a starry-sky appearance (×400). (**D**): Immunohistochemical analysis shows that the blasts are positive for lysozyme (×400). (**E**–**I**): Flow cytometric immunophenotypic analysis shows that the blasts are positive for CD34, CD117, CD4, CD33, CD38, CD64, CD123, HLA-DR, TdT, and MPO (dim, ~5%).

A 22-year-old woman presented with headache and a complete blood count (CBC) showed: WBC, 21.9 × 10^9^/L (reference range, 4–11 × 10^9^/L), hemoglobin, 8.3 g/dL (reference range, 14–18 g/dL), and platelets, 48 × 10^9^/L (reference range, 140–440 × 10^9^/L). A review of a peripheral blood smear showed 87% circulating blasts with monoblastic morphology characterized by open chromatin, variably conspicuous nucleoli, round to indented nuclear membranes, and moderate basophilic cytoplasm. No Auer rods were identified (Figure 1A). Computed tomography (CT) of the abdomen showed hepatomegaly (18.8 cm) and splenomegaly (16.4 cm) with no focal lesions. Bone marrow aspirate smears showed large blasts with morphologic features similar to the peripheral blood blasts (Figure 1B). The bone marrow biopsy specimen was hypercellular (100%) with sheets of immature cells (Figure 1C). Flow cytometry immunophenotyping performed on bone marrow aspirate detected blasts positive for CD34, CD117, CD33, CD38, TdT, HLA-DR, CD4, CD64, CD123 (increased), CD54 (partial), CD15, CD56, and MPO (dim, ~5% of cells) (Figure 1E–I) and were negative for CD13, CD19, surface and cytoplasmic CD3, and CD133. Immunohistochemistry performed on the bone marrow biopsy specimen showed that the blasts were positive for lysozyme (Figure 1D). These immunophenotypic findings support the diagnosis of acute monoblastic leukemia.

**Figure 2:**
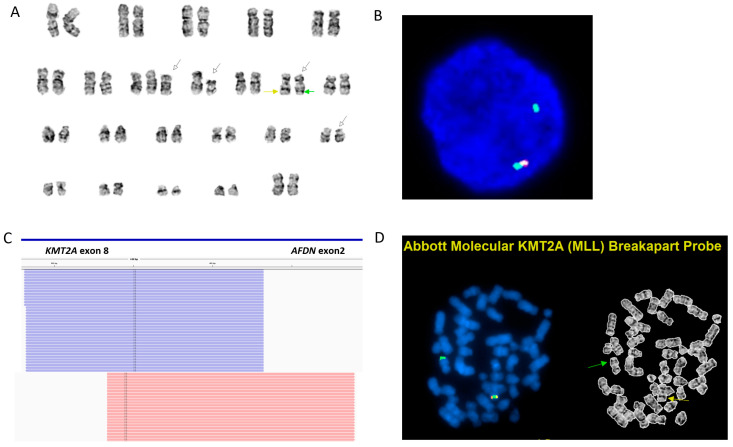
Chromosomal analysis, interphase FISH, RNA fusion panel, and metaphase FISH results. (**A**): Chromosomal analysis reveals a complex karyotype, 47,XX,+8,del(9)(q21q31)[11]/47,idem,inv(11)(q14q23)[8]. (**B**): Interphase FISH using a *KMT2A* break-apart probe shows one intact yellow fusion signal and one green signal, indicating a 3′ *KMT2A* deletion. (**C**): RNA fusion panel identifies fusion transcripts between exon 8 of *KMT2A* and exon 2 of *AFDN*. (**D**): Metaphase FISH reveals a normal chromosome 11 with a yellow fusion signal (yellow arrow) and a derivative chromosome 11 with only a 5′ *KMT2A* green signal (green arrow) and loss of 3′ *KMT2A*.

**Figure 3:**
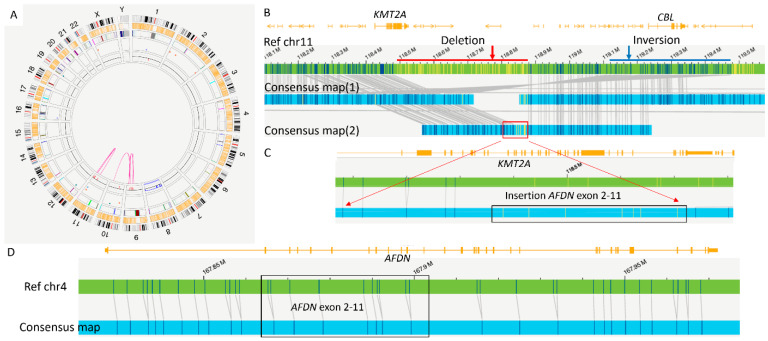
OGM results. (**A**) The OGM circos plot reveals trisomy 8, a deletion on the long arm of chromosome 9, and an interchromosomal translocation between chromosomes 9 and 10. Additionally, within chromosome 11q23, one inversion (marked by a blue dot), one deletion (marked by a red dot), and several intrachromosomal rearrangements are detected. (**B**) Initial review of OGM near the *KMT2A* locus highlights a 3′ end deletion of *KMT2A*, indicated by a thick red arrow and line on the reference chromosome 11, corresponding to the red dot in the circos plot (**A**). Furthermore, an inversion involving the *CBL* gene is denoted by a thick blue arrow and line on the reference chromosome 11, corresponding to the blue dot in the circos plot (**A**). (**C**) Detailed manual review of the insertion event reveals an insertion encompassing exons 2–11 of the *AFDN* gene, depicted by yellow bars within the red box on consensus map 2. This pattern of yellow bars matches that observed in (**D**). (**D**) Reference map and consensus map of chromosome 4 further illustrate the pattern of the *AFDN* (exon 2-11)). Throughout these figures, the OGM data are represented with specific visual elements to aid interpretation. The blue lines depict the alignment of the sample’s OGM map data (consensus map) to the reference genome. Within these blue lines, blue bars identify regions of consistent alignment or matched segments, while yellow bars pinpoint regions where structural variations—such as deletions or insertions—have been detected.

Conventional chromosomal analysis showed a complex karyotype: 47,XX,+8,del(9)(q21q31)[11]/47,idem,inv(11)(q14q23)[8] (Figure 2A). FISH for *KMT2A* showed deletion of *3′ KMT2A* in 95% of interphase nuclei, suggesting an unbalanced rearrangement involving *KMT2A* (Figure 2B). The Archer RNA fusion panel identified an in-frame *KMT2A::AFDN* fusion transcript involving exon 8 of *KMT2A* (NM_005933.3) and exon 2 of *AFDN* (NM_001291964.1) (Figure 2C). The initial OGM analysis using our routine “rare variants” automated bioinformatics pipeline provided additional insights, detecting multiple numerical and structural abnormalities, including +8, del(9q21.31q31.2)(79,486,359_107,159,358) x1, interchromosomal fusion t(9;10)(q32;q25.1)(114,326,068;104,682,589), inv(11)(q23.3q23.3)(118,321,159_119,475,489) involving *CBL*, del(11q23.3)(118,485,078_118,873,559) x1 affecting the 3′ end of *KMT2A*, and two intrachromosomal fusions: fus(11;11)(q14.3;q23.3)(90,516,821;120,252,883) and fus(11;11)(q23.3;q24.3)(119,344,090;129,505,513). However, there was no evidence of the canonical t(6;11) translocation (Figure 3A,B). To address these discrepancies, metaphase *KMT2A* FISH (break-apart fusion probe) was performed to assess whether *KMT2A* could be translocated to another chromosome, but the results did not support this possibility. The fusion signal was observed on the normal chromosome 11, and the 5′ *KMT2A* signal was localized to the derivative chromosome 11 (Figure 2D).

These results prompted a manual review of the OGM data subsequently, which identified a cryptic ∼300 kb insertion of *AFDN* (exons 2–11) into the 3′ region of *KMT2A* (exon 8) (Figure 3C,D). This finding corroborated the *KMT2A*::*AFDN* fusion transcripts detected by the RNA fusion panel, reconciling the discrepancies between chromosomal analysis/FISH and RNA fusion results.

## 3. Discussion

In this case, the cytogenetic and molecular findings were initially intriguing due to the complexity of the genomic event at 11q23. Conventional chromosome analysis did not detect the canonical balanced translocation t(6;11)(q27;q23), which results in *KMT2A::AFDN* fusion. Instead, interphase FISH identified a 3′-*KMT2A* loss, suggesting the presence of an unbalanced translocation. The RNA fusion panel detected the *KMT2A::AFDN* fusion transcript, providing transcriptional evidence of the rearrangement. However, metaphase FISH did not resolve the discrepancy between the cytogenetic findings and the RNA fusion results. This discrepancy prompted a manual review of the OGM data that initially identified an unknown insertion at the *3′-KMT2A* region. Upon detailed re-analysis, incorporating RNA fusion panel findings, we identified a cryptic insertion of *AFDN* into the 3′ region of *KMT2A* as a part of a complex genomic event on chromosome 11. This finding underscores the critical role of professional review in the OGM sign-out process and highlights the pitfalls of over-reliance on automated bioinformatics pipelines in analyzing complex genomic events.

To our knowledge, this is the second reported case of a cryptic insertion involving *AFDN* into the 3′-*KMT2A*, alongside one previously described case [12]. In the prior report, conventional karyotyping appeared normal, but further analysis using a whole chromosome 11 paint detected one *KMT2A::AFDN* fusion. An *AFDN* cosmid probe indicated that the other fusion resulted from the insertion of a submicroscopic portion of chromosome 6, including part of *AFDN*, into an apparently normal chromosome 11, a finding confirmed by RT-PCR [12]. Furthermore, three reports have described the insertion of *5′ KMT2A* into *AFDN*, with two of them being cryptic, leading to *KMT2A::AFDN* fusions [6,9,11]. In a study of 30 cases with *KMT2A::AFDN* fusion, there was only one case with insertion, highlighting its rarity [6].

Moreover, in a study of 183 hematologic malignancies with *t(4;11)/KMT2A::AF4*, presented at the European 11q23 Workshop, only five cases involved variant or complex translocations, none of which were insertions [13]. This suggests that while insertions represent an uncommon mechanism of *KMT2A* rearrangement, they are a recognized phenomenon.

Among insertion-induced *KMT2A* fusion events, insertions of the 5′ *KMT2A* segment into partner genes are more frequently observed than insertions of partner genes into the 3′ *KMT2A* segment (80% vs. 20%) [14]. Our case, involving the insertion of the partner gene *AFDN* into 3′ *KMT2A*, represents a rare event. Cryptic insertions like the one in our case evade detection by traditional cytogenetic assays, including chromosomal analysis and FISH, and may be underestimated in routine diagnostics [8,15]. RNA-based assays—such as RNA fusion panels or transcriptome sequencing—are often preferred for uncovering cryptic rearrangements resulting in fusion transcripts [15].

In AML with *KMT2A::AFDN*, breakpoints typically occur in intron 8 or intron10 of *KMT2A* and intron 1 of the *AFDN*, findings that align with the case we report [7]. The AML in the patient we report exhibited monoblastic differentiation, consistent with prior reports that AML with *KMT2A::AFDN* typically exhibits myelomonocytic or monocytic morphologic and immunophenotypic features. *KMT2A*-rearranged acute monoblastic leukemia is usually negative for CD34; however, this neoplasm case falls within the uncommon 5% of cases that demonstrate CD34 positivity [16].

Although t(6;11)/*KMT2A::AFDN* was the sole abnormality in two-thirds of cases reported, trisomy 8 is one of the most common additional abnormalities, with a frequency of 10% [6], as observed in this case. AML with *KMT2A::AFDN* is associated with a poor prognosis and a high risk of relapse, making the identification of this cryptic fusion critical for classification, risk stratification, and patient management.

This patient underwent induction chemotherapy with fludarabine, cytarabine, granulocyte colony-stimulating factor with idarubicin, cytarabine, and venetoclax (FLAG -IDA+VEN) and achieved complete remission with minimal residual disease negativity by flow cytometry, followed by consolidation therapy with FLAG-IDA+VEN. She is currently ~2 weeks status post allogeneic stem cell transplantation in first complete remission.

In summary, this case highlights both the promise and challenges of integrating novel molecular and genomic tools—such as RNA fusion panels and OGM—alongside traditional techniques such as chromosome analysis and FISH. While these new technologies have the potential to provide detailed characterization of genetic alterations at both the RNA and DNA level, enabling detection of cryptic abnormalities, their routine clinical application is not without limitations. Each assay has its own strengths and constraints, and assay discordance remains a key challenge in multi-modal testing. Discrepancies between methodologies can arise due to differences in sensitivity, coverage, or bioinformatic interpretation, necessitating careful evaluation by trained professionals to resolve inconsistencies. Additionally, the increased complexity of multi-modal testing requires expertise in integrating diverse genomic data, ensuring accurate classification, risk stratification, and prognostication of AML, ultimately leading to improved patient outcomes.

## Data Availability

Data are contained within the article.
